# The implementation in Europe (EU) of the drinking guidelines for the Early Identification and Brief Interventions (EIBI) for Hazardous and Harmful Alcohol Consumption (HHAC): results from the RARHA survey

**DOI:** 10.1186/1940-0640-10-S2-P16

**Published:** 2015-09-24

**Authors:** Emanuele Scafato, Ghirini Silvia, Gandin Claudia, Galluzzo Lucia

**Affiliations:** 1 Istituto Superiore di Sanità, , Italy

## Background

RARHA (REDUCING ALCOHOL RELATED HARM) is a Joint Action (JA) (2014-2016) funded under the EU Health Programme and by EU Member States to address some commonly identified priorities to reduce levels of alcohol related harm in the EU. The work package 5 contributes towards increased understanding among public health policy makers of the scientific basis and practical implications of the use of drinking guidelines as a public health measure, and towards more aligned messages to the general population, subgroups and health professionals.

The objectives are to evaluate the presence (or not) of guidelines on EIBI for HHAC on the basis of the existing and available EU projects/documents (PHEPA, ODHIN, BISTAIRS, WHO) (1-4) and by additional information based on ad hoc survey across European Union Member States.

## Material and methods

A country specific questionnaire has been developed by the National Observatory on Alcohol for confirming the available sources of EU projects or documents and for collecting and/or upgrading information on drinking guidelines used in the context of EIBI. The form has been submitted by email to the country representatives of the Committee on National Alcohol Policy and Action as members with qualified experience and competence. Participants have been asked to check the validity of the information provided by the country questionnaire reported as "review of available sources" and to provide the most updated and reliable information for their Country (The survey started on May 2014).

## Results

Twenty-nine out of 31 European countries selected (all RARHA associated and collaborating countries + 3 additional countries), replied to the RARHA questionnaire. Twenty countries have a governmental organization responsible for preparing clinical guidelines for managing HHAC; twenty-two countries have multidisciplinary guidelines approved or endorsed by at least one health care professional body or scientific society and 22 countries have guidelines or recommendations for BI/treatment.

## Conclusions

The collected information represent the scientific basis and practical implications of the use of drinking guidelines as a public health measure. It serves to clarify the science underpinnings and practical/policy implications concerning low risk drinking guidelines, and work towards consensus on good practice principles in the use of drinking guidelines as a public health measure to help reduce HHAC. This activity, finalized to provide a more aligned messages to the general population, subgroups and health professionals, is ongoing (Rarha Delphi survey).
